# "One‐Pot" Sample Processing Method for Proteome‐Wide Analysis of Microbial Cells and Spores

**DOI:** 10.1002/prca.201700169

**Published:** 2018-04-16

**Authors:** Bhagyashree Nandakishor Swarge, Winfried Roseboom, Linli Zheng, Wishwas R. Abhyankar, Stanley Brul, Chris G. de Koster, Leo J. de Koning

**Affiliations:** ^1^ Department of Mass Spectrometry of Bio macromolecules University of Amsterdam Amsterdam The Netherlands; ^2^ Department of Molecular Biology and Microbial Food Safety Swammerdam Institutes of Life Sciences, University of Amsterdam The Netherlands

**Keywords:** Mass spectrometry, One‐pot method, Proteomics, Spores, ZIC‐HILIC

## Abstract

**Purpose:**

Bacterial endospores, the transmissible forms of pathogenic bacilli and clostridia, are heterogeneous multilayered structures composed of proteins. These proteins protect the spores against a variety of stresses, thus helping spore survival, and assist in germination, by interacting with the environment to form vegetative cells. Owing to the complexity, insolubility, and dynamic nature of spore proteins, it has been difficult to obtain their comprehensive protein profiles.

**Experimental design:**

The intact spores of *Bacillus subtilis, Bacillus cereus*, and *Peptoclostridium difficile* and their vegetative counterparts were disrupted by bead beating in 6 m urea under reductive conditions. The heterogeneous mixture was then double digested with LysC and trypsin. Next, the peptide mixture was pre‐fractionated with zwitterionic hydrophilic interaction liquid chromatography (ZIC‐HILIC) followed by reverse‐phase LC‐FT‐MS analysis of the fractions.

**Results:**

"One‐pot" method is a simple, robust method that yields identification of >1000 proteins with high confidence, across all spore layers from *B. subtilis, B. cereus*, and *P. difficile*.

**Conclusions and medical relevance:**

This method can be employed for proteome‐wide analysis of non‐spore‐forming as well as spore‐forming pathogens. Analysis of spore protein profile will help to understand the sporulation and germination processes and to distinguish immunogenic protein markers.

## 1 Introduction

Proteomics techniques are widely used in clinical microbiology to analyze proteomes of complex pathogenic life forms, to investigate molecular mechanisms of pathogenesis, to identify biological markers, and to aid efficient clinical analysis. Gram‐positive spore formers such as *Bacillus anthracis* and *Clostridium tetani* are known to cause clinical infections. Spores of some *Bacillus* spp. are the causative agents of food spoilage and other food‐borne diseases,^1^ whereas spores of *Peptoclostridium difficile* play a critical role in *Clostridium difficile* infection (CDI), pseudomembranous colitis, and diarrhea. This imposes a high burden on the healthcare systems.^2^ Endospores formed by these pathogens are dormant, multilayered entities resistant to many physical stresses and chemicals. Their control is thus a challenge for food processing^3^ and healthcare industries.^4^ Spores can constantly sense the environment and in favorable conditions, they "return to life" rapidly by the process of "germination" and grow out. Once spore germination is triggered, it activates the downstream signaling pathways causing spore revival.^5^, ^6^, ^7^ Though spores themselves are not harmful, the vegetative cells that emerge from them can produce toxins and therefore, quick detection and removal of spores is important.

Spore structure is largely composed of proteins that are important for maintaining spore resistance. Furthermore, in sporulation, germination, and pathogenesis, proteins play important structural and functional roles. Therefore, a complete knowledge of the spore proteome is of utmost importance to gain insights into the missing links in both these processes. Unfortunately, the overall structure of the outer spore layers, that is, spore coat and/or exosporium, and the presence of an insoluble protein fraction therein (approximately 30%) makes a thorough spore protein analysis challenging. These unique proteinaceous and glycoproteinaceous structures protect the spores from different external stresses and also provide a mechanism to adhere to surfaces.^8^ A high‐throughput analysis, generally desired in clinical applications, is difficult owing to variation in sample quality, quantity, and biological heterogeneity among samples within a batch. Although different gel‐based and gel‐free methods coupled to mass spectrometry (MS) have been developed for identification and quantification of spore proteomes,^9^, ^10^, ^11^ most of these use detergents for spore disruption and protein solubilization. These detergents are mostly incompatible with MS analyses and therefore need to be removed before MS either by filtration, precipitation, or by gel‐based approaches. This extensive sample handling bears an increased risk of significant sample loss. Moreover, these methods are laborious and time consuming.^12^, ^13^ Therefore, to understand the mechanism of dormancy and that of exit from dormancy, there is a need for a method which will simultaneously identify proteins from all spore layers, including the insoluble protein fraction of spore surface layers. Moreover, the same method should be useful to uncover the proteome of the vegetative cell that emerges once the spores germinate.

**Clinical Relevance**

Mass‐spectrometry‐based proteomics is a powerful tool in the field of clinical microbiology. It helps in rapid detection of pathogens and identification of clinical protein markers, thereby improving therapeutic strategies. Though proteomics studies are now routine, studies for pathogenic spore formers belonging to the genera *Bacillus*, *Clostridium* have proven challenging, owing largely to the dimorphic nature of such species. Endospores, in general, are extremely resistant structures that survive the conventional inactivation and killing methods applied in the food and health industries. Moreover, in the presence of favorable conditions, spores can germinate and grow out via the process of "Germination." Additionally, the multilayered structure of the spore comprises proteins that play important roles in the structural integrity, germination, and pathogenesis. Various gel‐based and gel‐free approaches have been employed for protein identification from different spore layers. However, the insoluble protein fraction within these outer layers proves another hurdle for comprehensive proteomic characterization. We present a novel method to identify soluble and insoluble proteins from spores in a single pot. The extendibility of "one‐pot" method to a variety of pathogenic species is demonstrated here using *Bacillus subtilis*, *Bacillus cereus*, and *Peptoclostridium difficile*.

**Figure 1:**
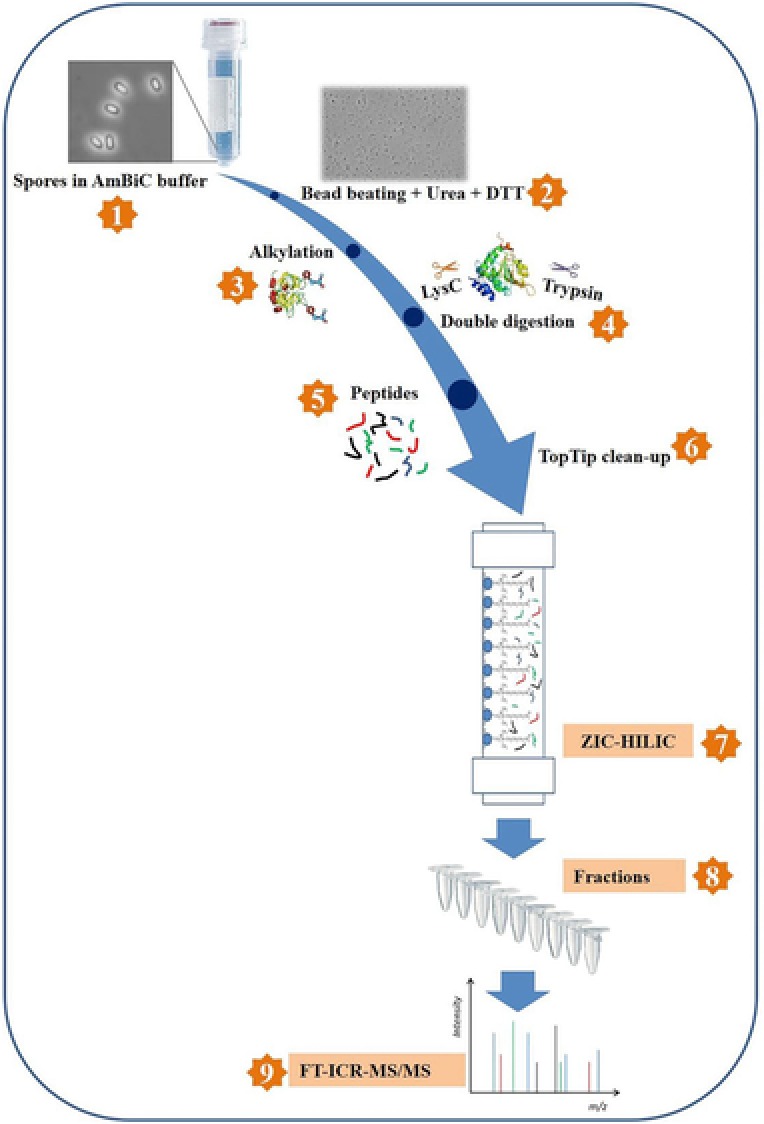
Schematic representation of "one‐pot" sample processing method. Steps 1–5 are performed in a single tube.

Generally, complex peptide mixtures necessitate pre‐fractionation by multidimensional approaches prior to MS analysis.^14^ zwitterionic hydrophilic interaction liquid chromatography (ZIC‐HILIC)‐based fractionation coupled with reverse‐phase LC‐FTICR‐MS has been developed as a powerful tool for such proteomic analyses.^15^, ^16^ In our "one‐pot" sample processing method (Figure 1), proteins from whole spores are solubilized with the help of bead beating in the presence of urea and DTT, alkylated, and finally digested with LysC and trypsin in a single tube. We used *Bacillus subtilis* as a model organism to check the efficiency and robustness of the method. We also focused on two clinically relevant bacterial spore formers—*Bacillus cereus* and *P. difficile*. In this study, we successfully identified over 1000 proteins from the whole spores of these organisms. We have also extended the method to explore the vegetative cell proteome of these spore‐forming bacteria to evaluate how wide the scope of this method is, for other spore‐forming and non‐spore‐forming pathogens.

## 2 Experimental Section

### 2.1 Media, Strains, and Spore Preparation

The strains used in this study were *B. subtilis* PY79*, B. cereus* ATCC 14579, and *P. difficile* 630. The growth, sporulation, and spore harvesting for *B. subtilis* and *B. cereus* were carried out as described previously.^17^ *P. difficil*e 630 was acquired from the Leibniz Institute of Microorganisms and Cell Cultures. Growth of *P. difficile* 630 cells and spore preparations were done in an anaerobic chamber (Whitley DG250) aerated with a gas mixture (10% hydrogen, 10% carbon dioxide, and 80% nitrogen), with the temperature controlled at 37 °C. *P. difficile* cells were harvested from a culture in Schaedler Anaerobe Broth (Oxoid) at mid‐log phase (OD_600_ = ≈0.7) and spores were obtained in CloSpore medium, harvested ,and purified, as described previously.^18^ More than 95% phase bright spores, as observed with phase contrast microscopy, were obtained for all the three organisms. Three biological replicates for *B. subtilis* PY79 spores and two each for *B. cereus* ATCC 14579 and *P. difficile* 630 spores were analyzed. One replicate each of *B. subtilis* PY79 and *P. difficile* 630 vegetative cells was also analyzed.

### 2.2 "One‐Pot" Sample Processing Method

Phase bright spores of both the bacilli and the *Peptoclostridium* sp. were suspended in lysis buffer containing 6 m urea, 5 mm DTT in 50 mm ammonium bicarbonate (AmBiC) buffer (pH = 8.0) and disrupted with 0.1 mm zirconium‐silica beads (BioSpec Products, Bartlesville, OK, USA) using a Precellys 24 homogenizer (Bertin Technologies, Aix en Provence, France). A *B. subtilis* spore sample without urea was also used as a control to check effect of urea on protein extraction. Spores were disintegrated for seven rounds (each round of 20 s, 60 s pause between each round). Samples were placed on ice for 10 min after every three rounds to avoid protein degradation by overheating. To check the effect of bead beating on proteins, a protein kit containing bovine serum albumin (BSA), myoglobin, β‐casein, and horse cytochrome C was used as bead‐beating control. The total amount of protein material extracted from spore was estimated using the reducing‐agent‐compatible version of bicinchoninic acid (BCA) Protein Assay Kit (Thermo Fisher Scientific, Waltham, MA, USA). The reduction was carried out at 56 °C for 1 h followed by alkylation by 15 mm iodoacetamide (IAA) for 45 min at room temperature in dark. The reaction was quenched with 20 mm thiourea.^19^ Samples were then digested with LysC (1:200 w/w protease/protein) for 3 h at 37 °C. Samples were diluted with 50 mm AmBiC and 20% ACN followed by digestion with trypsin (1:100 w/w protease/protein ratio) at 37 °C for 18 h. The digestion reaction was quenched with the addition of TFA (pH < 4). All these steps were carried in a single tube and post digestion cell/spore debris was removed by centrifuging for 15 min at 13 000 rpm. The supernatant containing peptides was transferred to a new tube and freeze dried (if necessary). This tryptic digest was redissolved in 0.1% TFA, cleaned up using C18 reversed‐phase TT2 TopTips (Glygen), according to the manufacturer's instructions and peptides were eluted with 0.1% TFA in 50% ACN and freeze dried.

### 2.3 ZIC‐HILIC‐Based Peptide Fractionation

The freeze‐dried peptides were diluted with Buffer A for subsequent ZIC‐HILIC separation. The Buffer A contained 85% ACN, 5 mm ammonium acetate, and 0.4% acetic acid (pH 5.8) and Buffer B contained 30% ACN, 5 mm ammonium acetate, and 0.5% acetic acid (pH 3.8). Isocratic flow for 10 min with 100% Buffer A and elution was achieved with gradient of 0–30% of Buffer B in first phase and then 30–100% in second phase with the flow rate of 400 μL min^−1^. Eluted peptides were collected in 10 fractions, which were freeze dried prior to MS/MS analysis. The total amount of peptide material extracted from spore was estimated using the BCA Protein Assay Kit (Thermo Fisher Scientific, Waltham, MA, USA).

### 2.4 LC−FT‐ICR MS/MS Analysis

HILIC fractions were dissolved in 0.1% TFA and peptide concentration was determined at 205 nm^20^ with a Nanodrop ND1000 spectrophotometer (Isogen Life Sciences, De Meern, The Netherlands). LC‐MS/MS data were acquired with a Bruker Apex Ultra FT‐ICR mass spectrometer (Bruker Daltonics, Bremen, Germany) equipped with a 7 T magnet and a Nano electrospray Apollo II Dual Source coupled to an Ultimate 3000 (Dionex, Sunnyvale, CA, USA) UPLC system. Samples containing up to 300 ng of the tryptic peptide mixtures were injected as a 40 μL 0.1% TFA, 3% ACN aqueous solution with 1 μL of 50 fM [Glu1]‐Fibrinopeptide B, as an internal standard, and loaded onto a PepMap100 C18 (5 μm particle size, 100 Å pore size, 300 μm inner diameter × 5 mm length) precolumn. Following injection, the peptides were eluted at 30 °C via an Acclaim PepMap 100 C18 (5 μm particle size, 100 Å pore size, 75 μm inner diameter × 500 mm length) analytical column (Thermo Fisher Scientific, Etten‐Leur, The Netherlands) to the nano‐electrospray source. Gradient profiles of up to 140 min were used from 0.1% formic acid/3% ACN/97% H_2_O to 0.1% formic acid/50% ACN/50% H_2_O at a flow rate of 300 nL min^−1^. Data‐dependent Q‐selected peptide ions were fragmented in the hexapole collision cell at an argon pressure of 6 × 10^−6^ mbar (measured at the ion gauge) and the fragment ions were detected in the ICR cell at a resolution of up to 60 000. In the MS/MS duty cycle, four different precursor peptide ions were selected from each survey MS. The MS/MS duty cycle time for one survey MS and three MS/MS acquisitions was about 2 s. Each MSMS dataset was mass calibrated internally on the [Glu1]‐Fibrinopeptide B peptide fragment ion masses better than 1.5 ppm over a *m*/*z* range of 250–1400.

### 2.5 Data Analysis

Raw FT‐MS/MS mass calibrated data of the HILIC fractions was processed as multi‐file (MudPIT) with the MASCOT DISTILLER program, version 2.4.3.1 (64 bits), MDRO 2.4.3.0 (MATRIX science, London, UK). Peak‐picking for both MS and MS/MS spectra were optimized for the mass resolution of up to 60 000 (m/Δm). Peaks were fitted to a simulated isotope distribution with a correlation threshold of 0.7, and with a minimum signal‐to‐noise ratio of 2. The processed data, combined from the ten HILIC peptide fractions, were searched in a MudPIT approach with the MASCOT server program 2.3.02 (MATRIX science, London, U.K.) against a complete *B. subtilis* 168, *B. cereus* ATCC 14579, and *P. difficile* 630 ORF translation database (Uniprot 2017‐02‐13 update, downloaded from http://www.uniprot.org/uniprot) with redundancies removed using the DBToolkit‐4.2.5 tool (http://bioinformatics.oxfordjournals.org/cgi/reprint/bti588?ijkey=1d1b7RussnjgEkC&keytype=ref) and supplemented with the corresponding decoy database to determine FDR. Trypsin was used as the enzyme and two missed cleavages were allowed. Carbamidomethylation of cysteine was used as a fixed modification and oxidation of methionine and deamination of asparagine and glutamine as variable modifications. The peptide mass tolerance and peptide fragment mass tolerance were set to 50 ppm. The search was repeated with the same parameters but with semi‐trypsin as the enzyme to identify possible semi‐tryptic peptides due to mechanical shearing and possible endogenous degradation of proteins. The MASCOT MudPIT peptide identification score was set to a cutoff of 20 with a false discovery rate of approximately 2% at the peptide level based on decoy database matches. The identification data are listed in Table 1, Supporting Information. The raw proteomics data has been deposited to the Proteome change Consortium^21^ via the PRIDE partner repository with the dataset identifier PXD008242.

### 2.6 Bioinformatics Analyses

Being clinically relevant species, efforts were put in the bioinformatics analyses of the identified proteins from spores of *B. cereus* and *P. difficile*. We analyzed the immunogenic potential of the identified peptides using the automated algorithm at the POPI v.2.0 server.^22^ Functional annotation clustering of identified proteins from all the three spore formers was carried out with the help of tools available at DAVID bioinformatics resources.^23^, ^24^ Domain predictions were done using the Batch CD‐search tool developed by the National Center for Biotechnology Information (NCBI).^25^ The molecular mass (kDa), p*I*, and GRAVY indices of proteins were established using the ProtParam tool.^26^

## 3 Results and Discussion

### 3.1 "One‐Pot" Sample Processing Validation

The one‐pot sample processing is started with a physical disruption of the spores and bacterial cells by a number of bead‐beating cycles until no intact spores and cells are microscopically observed. This bead‐beating disruption is prone to cause thermal degradation of the proteins.^26^ Such degradation results in the formation of semi‐tryptic peptides during tryptic digestion. For a protein kit with BSA, myoglobin, cytochrome‐C, and β‐Casein the one‐pot processing yields an insignificant contribution of semi‐tryptic peptides if the bead‐beating event is omitted both in the presence and absence of urea. With bead beating this contribution is raised to about 10% in the presence of urea for the mixture containing *B. subtilis* spores and the protein kit, and further to over 20% when the urea is omitted. This is observed for both spore proteins as well as the peptides originating from the protein kit. Although the abundances of the semi‐tryptic peptides are low relative to the tryptic peptides, it is clear that bead beating causes some degradation and necessitates proper cooling of the samples between various bead‐beating cycles. Though this degradation has no effect on protein identification, it undesirably increases the complexity of the digest mixture. Thus to minimize this, proteolytic enzyme activities of spore proteins are quenched through the inclusion of urea in the mixture. Consequently, it increases the average peptide sequence coverage of the proteins identified in *B. subtilis* spores from 19% to 27%. For a one‐pot processing it is also critical to quench the excess DTT and the cysteine alkylating IAA before digestion to prevent overalkylation and alkylation of peptide N‐termini. The applied quenching with thiourea is efficient and yields quenching products which do not affect the protein digestion.^19^

To allow peptide extraction from the disrupted spores under 6 m urea protein denaturing conditions, digestion is initiated through cleavage of the lysine residues by LysC. Thereafter, dilution of the digest buffer to 1 m urea enables the remaining lysine and arginine residues to be cleaved by trypsin to bring the peptide masses within the mass window of the mass spectrometric analysis. Quenching the tryptic digestion with TFA brings down the pH to below 4. Under these acidic conditions most of the fatty acids, lipids, and peptidoglycans precipitate and are removed by centrifugation together with the remaining cell debris before freeze drying. The low pH initiates the hydrolysis of the phosphodiester backbone of DNA and RNA. Cleanup of the freeze‐dried supernatant with a C18 TopTips removes such remaining digestion buffer components and reactants. Finally, the residual nonpeptide material is not retained and washed off the ZIC‐HILIC column resulting in ZIC‐HILIC HPLC peptide fractions ready for reverse‐phase LC‐MS/MS analysis. As estimated by the BCA assay, about 630 μg of protein material is extracted from 1 mg of *B. subtilis* spores and about 60 μg is recovered as peptides after ZIC‐HILIC pre‐fractionation, before mass spectrometric analysis. The one‐pot processing is, though protein recovery is ≈10%, more efficient than other currently available strategies.

### 3.2 The Vegetative Cell and Spore Proteome Coverage

**Figure 2:**
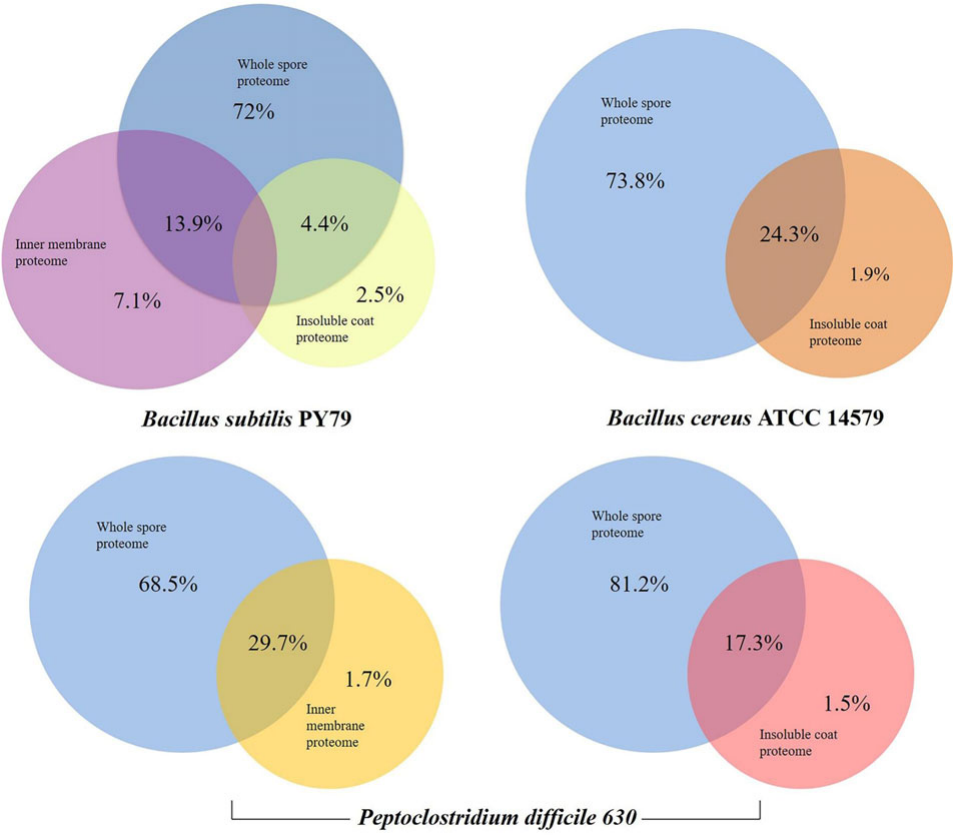
Comparison of protein identification by "one‐pot" method and conventional gel‐free methods. For comparison, the datasets obtained by gel‐free protein identification of insoluble coat fractions^12^, ^17^ and spore inner membrane proteomics using membrane enrichment approach^27^ are used.

From the intact‐spore extracts of *B. subtilis*, *B. cereus*, and *P. difficile*, 1428, 1782, and 1078 proteins have been identified, respectively ( Table 1, Supporting Information), with average peptide sequence coverage over all replicates being 26.6%, 20.3% and 15.2%, respectively (Figure 1, Supporting Information). From the *B. subtilis* and *P. difficile* vegetative cells, 1258 and 962 proteins have been identified, respectively. It appears that the relatively low LC‐MS/MS rate of the used mass spectrometer is limiting the number of identified proteins. The LC‐MS survey analyses show complex peptide mixtures of which only a limited number can be MS/MS analyzed. This together with the wide molecular mass distribution for identified proteins (Figure 2, Supporting Information) demonstrates the potential of the present one‐pot sample processing method for comprehensive proteome‐wide analysis of pathogens using high MS/MS rate mass spectrometers. A functional annotation clustering of the identified spore proteins which may be relevant for the spores and spore germination for the three organisms is shown in Table 1.

**Table 1:** A functional annotation clustering of identified proteins from whole spores of *Bacillus subtilis*, *Bacillus cereus*, and *Peptoclostridium difficile* as classified using the UP_Keyword and KEGG_pathway annotation available at the DAVID functional annotation tool

Functional annotation cluster	*B. subtilis* PY79	*B. cereus* ATCC14579	*P. difficile* 630
**UP_Keyword**			
Membrane	243	251	135
Cytoplasm	279	211	154
Sporulation	130	20	47
Hydrolase	219	229	147
Oxidoreductase	159	177	75
Transferase	205	261	155
ATP binding	186	174	123
Protein synthesis	38	41	37
Protease	51	44	28
Chaperons	18	13	11
Stress response	43	11	9
**KEGG pathway**			
Glycolysis/gluconeogenesis	32	39	19
TCA cycle	22	24	6
Biosynthesis of amino acids	65	81	55

The numbers represent the identified proteins in the category.

### 3.3 Comparison with Prevailing Gel‐Free Sample Preparation Methods

The gel‐free methods of spore proteomics previously developed in our lab were confined to identification of proteins from specific layers.^12^, ^27^ We compared the results of the "one‐pot" sample processing method (Figure 2) with the previous data obtained from spores of *B. subtilis*,^12^ *B. cereus*, and *P. difficile* 17 and also with the inner membrane protein data for *B. subtilis* 27 and *P. difficile* (unpublished data). Although the methods described by Kuwana et al.^28^and Mao et al.^29^ focus on the whole spore proteomes, they cannot be directly compared to "one‐ pot" method owing to the differences regarding the use of gels, sample processing, MS instruments, and data analysis. Similarly, Lawley and coworkers^30^ identified proteins from whole spores of *P. difficile*. However, a major hurdle of their method is that it involves multiple steps which make the entire procedure rather cumbersome and prone to sample loss. While we are positive of our current results, some membrane proteins could not be identified by this method. Of these unidentified proteins from *B*. *subtilis* and *P. difficile*, most showed peptide scores less than 20 and therefore, are not discussed here. This outcome can be improved by employing enrichment methods such as subcellular fractionations.^31^

### 3.4 Physicochemical Properties of Identified Spore Proteins

**Figure 3:**
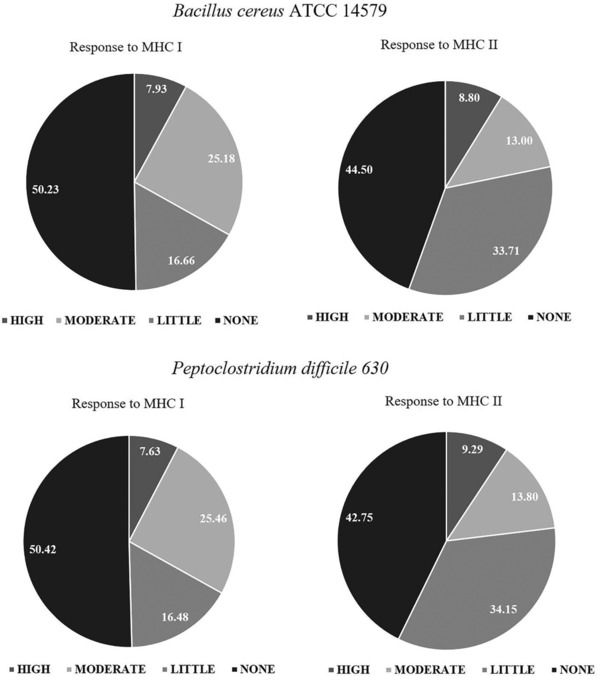
Prediction of immunogenic potential of identified peptides from *Bacillus cereus* ATCC 14579 and *Peptoclostridium difficile* 630. The MHC responses are classified as None (PD_50_ > 10 mm), Little (PD_50_ = 10 mm–100 nm), Moderate (PD_50_ = 100–1 nm) or High (PD_50_ < 1 nm). PD_50_: the dosage that protects 50% of the animals challenged.

Interestingly, most of the identified spore proteins from the three species are hydrophilic and <100 kDa in size with some exceptions (Figure 3, Supporting Information). The mean GRAVY index is −0.297 for *B. subtilis*, −0.236 for *B. cereus*, and −0.237 for *P. difficile*. The surface layers of spores are said to be hydrophobic but the spore proteins identified in this study are hydrophilic. Thus it is plausible that the spore surface proteins along with the other reported components such as lipids, phosphorous, sugar moieties^32^, ^33^ impart hydrophobicity to the spore surfaces. To investigate the role of the identified proteins in pH‐dependent adhesive properties, we determined their p*I* values. The hydrophilic proteins were spread over both acidic and basic pH ranges. The mean p*I* of the identified *B. subtilis* spore proteome is 6.33 whereas those of *B. cereus* and *P. difficile* proteomes are 6.32 and 6.22, respectively. The p*I* values for all three organisms show unimodal distribution (see Figure 3, Supporting Information).

### 3.5 Identification of Potential Protein Targets from Spores

We identified coat‐ and exosporium‐associated and putative target proteins in *B. cereus* ATCC 14579 and in *P. difficile* 630 spores (see Table 1, Supporting Information) that were also identified previously.^17^ In addition, from *P. difficile* spores, we identified proteins GerG (CD0311), CD3298, and CD0727 that were the focus of recent functional studies.^34^, ^35^, ^36^ In a previous study, domain prediction approach identified proteins with domains predicted to be pathogenically critical and thus clinically relevant.^37^ From *P. difficile* and *B. cereus* spores, we identified proteins, from different spore layers, carrying the pathogenically critical superfamily domains such as AAA superfamily, Actin, ChtBD3, CTD, FtsZ, HAMP, HisKA, HTH_MARR, OmpH, TPK_B1_binding, Tryp_SPc, Tubulin, and YARHG domain (see Table 2, Supporting Information). Specificities on the functions of these superfamily domains are reviewed by Patel.^37^ Apart from these proteins, there were many proteins carrying domains of unknown function (DUFs). Based on a previous study,^38^ we identified the domains that are essential (eDUFs) for bacteria and those that belong to the top 50 DUFs (see Table 3, Supporting Information), thus generating a list of potential candidate proteins.

Protein CD2635 identified is a virion protein. This protein is downregulated in a σ^G^ mutant and suggested to be involved in spore germination^39^ making it a key candidate for *P. difficile* germination studies. In a recent proteomic study on spore assembly in *Clostridium perfringens*, protein cyanophycin synthase encoded by *cphA* was identified as an important structural component of spore coat.^40^ Its ortholog protein encoded by MurE (CD2664) in *P. difficile* was identified in our proteome set. The role of MurE in spore assembly remains unstudied. Protein CD3559 (FtsH2) is an ortholog of SpoVK,^41^ a protein involved in the engulfment process during spore formation in *B. subtilis*.^42^ Proteins CD3306 and BC_4480 (Tig) are trigger factor proteins, which aid the initial folding steps during protein synthesis and, together with ribosomes, interact with almost all synthesized nascent chains.^43^ Some t‐RNA processing enzymes, viz., CD3560 (TilS), CD3675, BC_5485 (MnmG), and CD3676 and BC_5486 (MnmE) were identified in spores. These belong to the synthetase and GTPase families, and are associated with modifying the wobble uridine base in tRNA anticodons.^44^, ^45^, ^46^ Saujet and colleagues have previously provided guidelines about the sigma factors that regulate the expression of genes related with sporulation in *P. difficile* 630.^39^ Compared to those guidelines, our study has identified 10 σ^F^‐regulated, 6 σ^G^‐regulated, 21 σ^E^‐regulated, and 11 σ^K^‐regulated proteins. Apart from these, six and four protein‐encoding genes were found to be σ^EFK^‐regulated and σ^EFG^‐regulated, respectively.

### 3.6 Immunogenicity Predictions for the Identified Peptides

In *B. cereus* and *P. difficile*, most of the identified peptides were predicted to have none or little immunogenic potential toward cytotoxic (MHC I) and helper (MHC II) T‐cells; however, ≈0.5% of the identified unique peptides were predicted to be highly immunogenic (Figure 3). Interestingly, a single peptide ‐KTIEDAMVSDK‐ from GerD (BC_0169) was identified to be highly immunogenic (PD_50_ < 1 nm) toward both MHC molecules. Similarly, for *P. difficile* 630, peptides ‐SLEEVESIK‐ and ‐LVDEDMAMK‐ from membrane proteins CD1788 and CD2056, respectively, ‐RFTEALDKK‐ from spore coat protein SipL, and ‐LEELKESAPSLSAEELK‐ from putative sporulation protein CD2717 were all identified to possess high (PD_50_ < 1 nm) immunogenic potential against both the MHC classes.

## 4 Concluding Remarks

With minimum sample loss and few processing steps, the "one‐pot" sample processing method is tailored for proteome‐wide characterization of bacterial pathogens. The method enables identification and quantification of the proteins over all spore and vegetative cell layers and therefore can be readily applied to any time‐series‐based proteomic analysis including vegetative growth, sporulation, and spore germination. Although vegetative cells have not been the focus of the present study, the sample processing method facilitates high‐throughput analyses of rapidly changing protein dynamics in actively growing vegetative cell cultures exposed to varying environmental conditions. It can be applied in industrial pharmaceutical studies as rapid and robust sample preparation for testing the potential of antimicrobial agents to control bacterial infections. Finally, the "one‐pot" sample processing method enables rapid and reliable identification of the bacterial pathogen as spores from food samples.

**Abbreviations**
AmBiC: ammonium bicarbonate
BCA: bicinchoninic acid
CD: conserved domains
DUFs: domains of unknown function
IAA: iodoacetamide
MDRO: MASCOT data reduction object
NCBI: National Center for Biotechnology Information
UPLC: ultra high pressure liquid chromatography
ZIC‐HILIC: zwitterionic hydrophilic interaction liquid chromatography

## Conflict of Interest

The authors declare no conflict of interest.

## Supporting information

Supporting Information

Supporting Information

Supporting Information
