# Supplementary material for: "One‐Pot" Sample Processing Method for Proteome‐Wide Analysis of Microbial Cells and Spores
Source: Proteomics Clin Appl. 2018 Apr 16;12(5):1700169. doi: 10.1002/prca.201700169 (PMC6174930; doi:10.1002/prca.201700169)
Supplement: Supplementary file 1 — Supporting Information [file PRCA-12-na-s001.docx]

**Supplementary Figures**

**
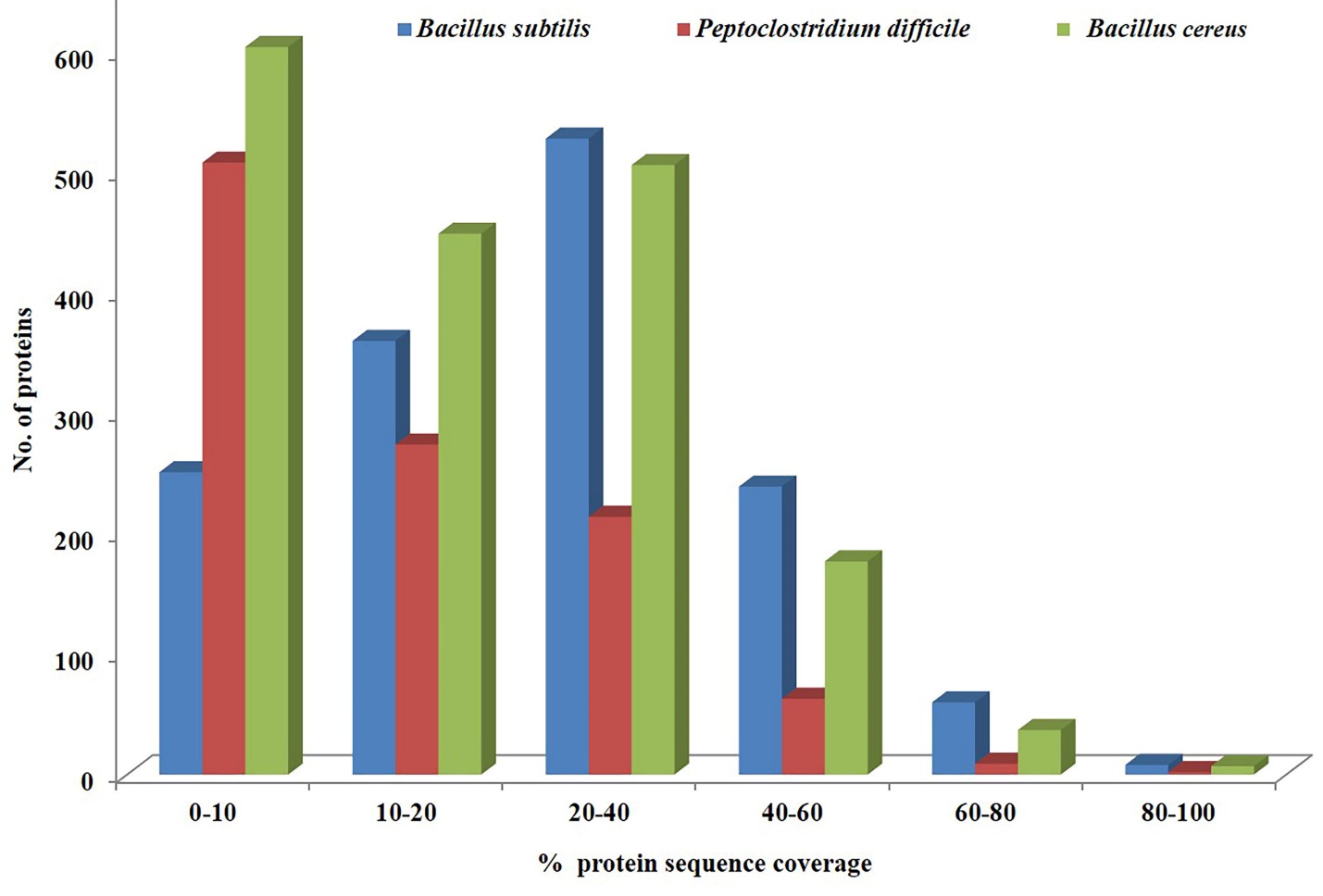
**

**Supplementary Fig.1. Overall protein sequence coverage of identified proteins from spores of *B. subtilis* PY79, *B. cereus* ATCC 14579 and *P. difficile* 630.** For *B. subtilis* most of the protein have sequence coverage more than 10% whereas for *B. cereus* and *P. difficile* maximum proteins have sequence coverage less than 10%.

**
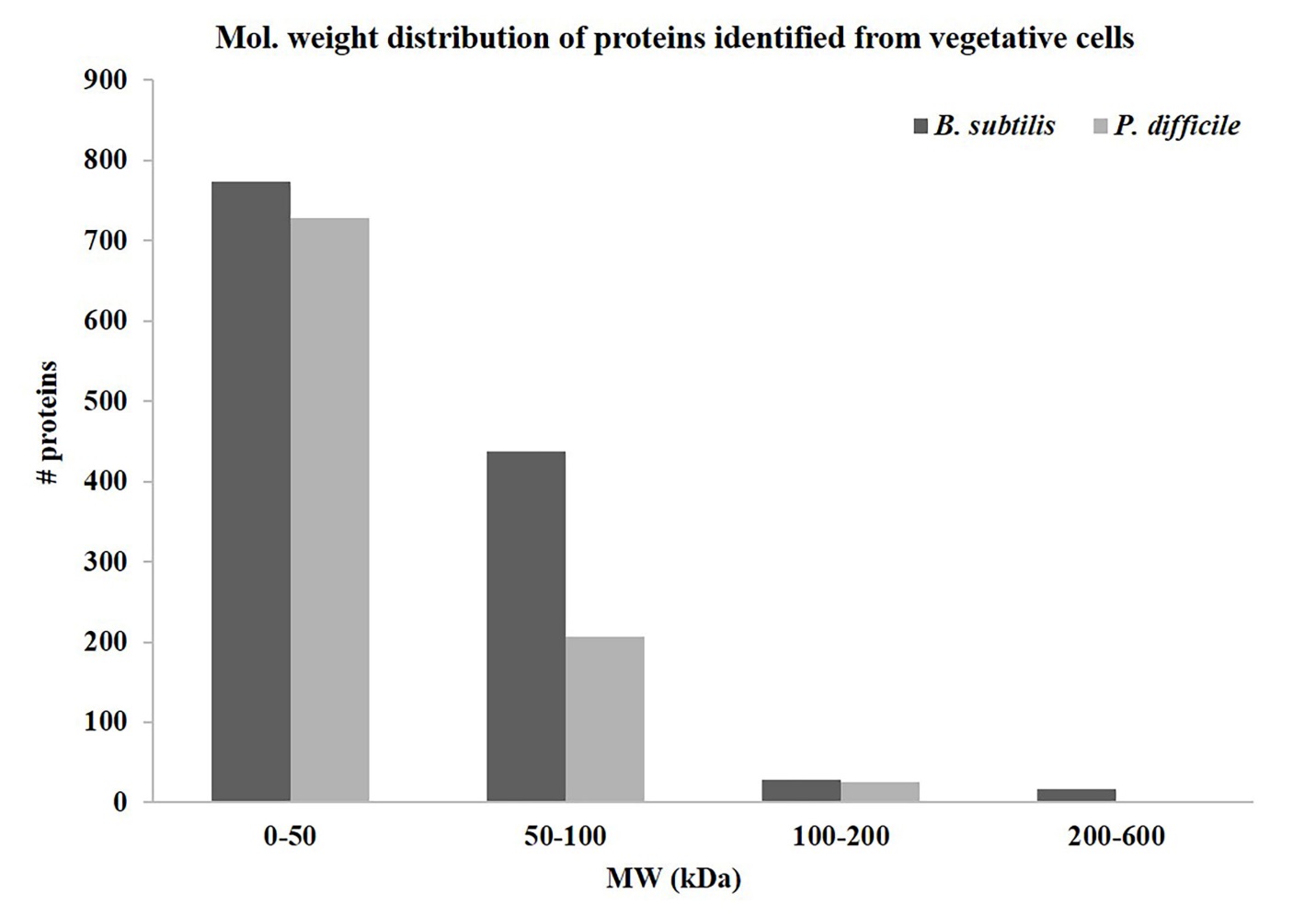
**

**Supplementary Fig. 2. Distribution of identified proteins from vegetative cells of *B. subtilis* PY79 and *P. difficile* 630 with respect to their molecular weights.**


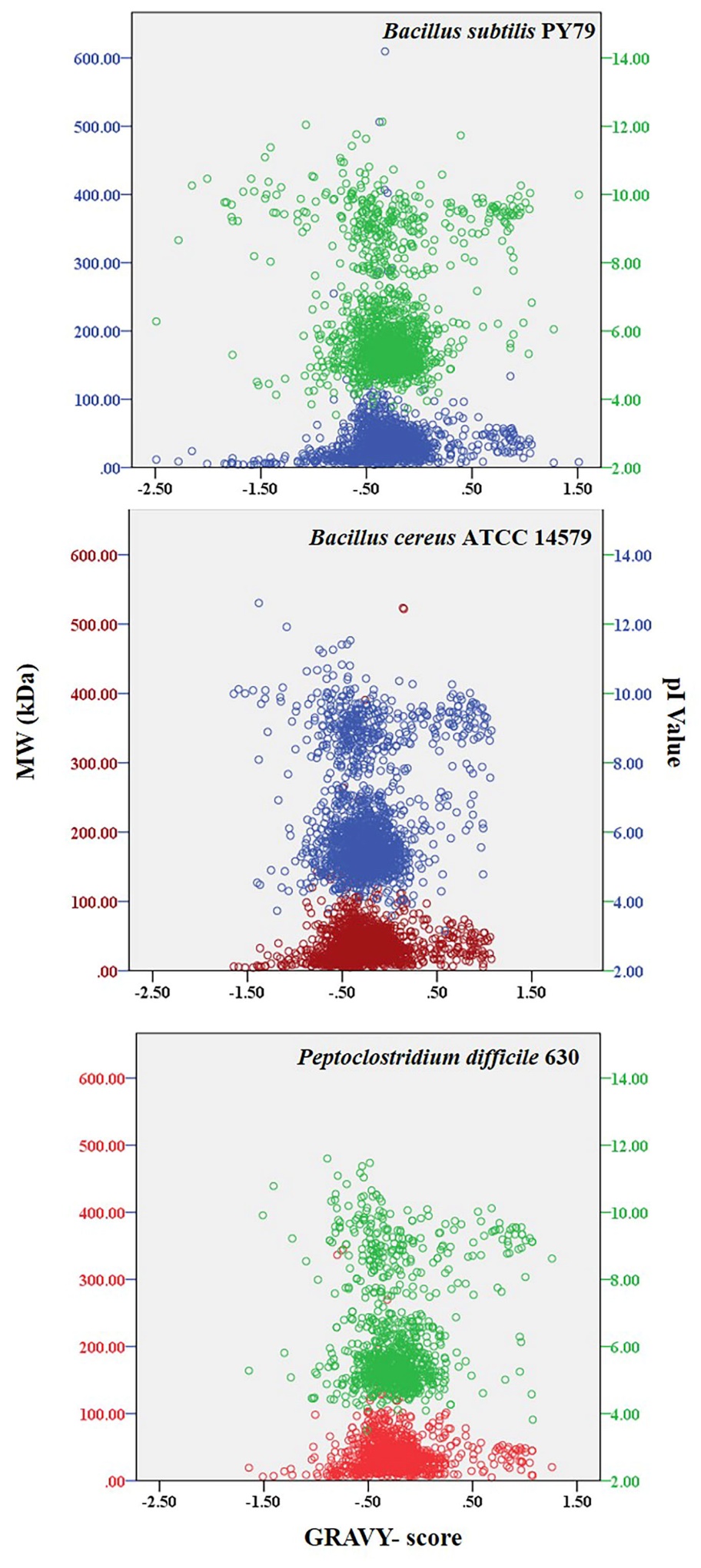


**Supplementary Fig. 3. Plot of molecular weight and p*I* versus GRAVY-score.** The p*I* values and molecular mass are plotted against GRAVY –scores of identified proteins of *B. subtilis* PY79, *B. cereus* ATCC 14579, *P. difficile* 630.

**Supplementary Tables**

**Supplementary Table 1. Identified proteins from spores of *B. subtilis*, *B. cereus* and *P. difficile* in this study.** Proteins identified in vegetative cells are also shown.

**Supplementary Table 2. Identified proteins containing the clinically relevant or pathogenically critical domains.**

**Supplementary Table 3. Identified proteins and their Domains of Unknown Functions (DUFs).**
